# Sugammadex: Safety Profile and Adverse Effects in Infants Less Than Two Years of Age With Congenital Heart Disease

**DOI:** 10.14740/cr2243

**Published:** 2026-07-17

**Authors:** Zachary Holtz, Stephania Paredes Padilla, Sibelle Aurelie Yemele Kitio, Julie Rice-Weimer, Joseph D. Tobias

**Affiliations:** a The Ohio State University College of Medicine, Columbus, OH, USA; b Department of Anesthesiology and Pain Medicine, Nationwide Children’s Hospital, Columbus, OH, USA; c Department of Anesthesiology and Pain Medicine, The Ohio State University College of Medicine, Columbus, OH, USA

**Keywords:** Sugammadex, Congenital heart disease, Adverse effects, Bradycardia

## Abstract

**Background:**

Despite widespread clinical use and Food and Drug Administration (FDA) approval for use in patients from birth to < 2 years old in 2024, data regarding the safety and monitoring practices of sugammadex in infants with congenital heart disease (CHD) remains limited. While its efficacy has been demonstrated, adverse effects have been documented including bradycardia and allergic reactions, including anaphylaxis. These effects may be of particular concern in infants with CHD, who may be at a higher risk for hemodynamic instability.

**Methods:**

The current study retrospectively evaluated a random sample of patients over a 10-year period to determine the efficacy, perioperative adverse effects, and neuromuscular monitoring practices associated with the administration of reversal agents for neuromuscular blocking agents in patients less than 2 years of age with CHD.

**Results:**

The study cohort included 497 encounters across 375 unique patients with a median age of 7 months who received either sugammadex or neostigmine, which served as a historical comparator. Bradycardia occurred at a low and comparable rate in both groups (1.4%), and no cases of anaphylaxis were documented. Additional adverse events were heterogeneous and were likely attributable to the underlying CHD, associated comorbidities, and the hemodynamic complexity of the surgical procedures rather than to the reversal agent alone. Successful tracheal extubation was achieved in over 90% of encounters, with a similar rate between groups. Rate of residual neuromuscular blockade was also comparable between groups, though quantitative train-of-four (TOF) monitoring was inconsistently documented. Furthermore, wide variability in sugammadex dosing was observed, highlighting an opportunity for standardization in this high-risk pediatric population.

**Conclusions:**

In the current cohort of infants with CHD, sugammadex was associated with a low incidence of perioperative adverse effects and rates of successful tracheal extubation similar to neostigmine. These patterns held despite higher ASA status among patients receiving sugammadex, which suggests greater baseline illness severity. Observed adverse events were heterogeneous and likely reflect the underlying clinical context in addition to any reversal agent effects. This study also highlights practice variability, including inconsistent documentation of quantitative TOF monitoring, which limits the ability to assess residual blockade and may contribute to variable dosing of sugammadex. In this physiologically vulnerable population, standardized monitoring and prospective data would help clarify how to best dose sugammadex. Taken together, these findings support the continued use of sugammadex in infants with CHD while highlighting the limits of current evidence. Larger, prospective studies with consistent monitoring are needed to better define the safe use of sugammadex in this population and to more clearly separate drug-related effects from other perioperative contributions to adverse events.

## Introduction

Unlike traditional acetylcholinesterase inhibitors such as neostigmine, which function to increase the acetylcholine concentrations at the neuromuscular junction to reverse neuromuscular blockade, sugammadex (Bridion^®^, Merck & Co, Whitehouse Station, NJ) works through a different mechanism. It is a modified γ-cyclodextrin that encapsulates the rocuronium or vecuronium molecule, forming a stable, one-to-one complex that prevents their action at nicotinic receptors on the sarcolemma [1–4]. By reducing the concentration of active neuromuscular blocking agents (NMBAs), sugammadex effectively leads to the rapid and complete reversal of neuromuscular blockade, even in the setting of deep or complete blockade [5, 6]. This unique mechanism eliminates the need for adequate spontaneous neuromuscular recovery prior to reversal and reduces the risk of residual neuromuscular blockade, which can contribute to postoperative respiratory complications [7].

Sugammadex was approved by the United States Food and Drug Administration (FDA) in December 2015 for use in adult patients undergoing surgery with an expanded indication to include pediatric patients ≥ 2 years of age in 2021. Throughout this time, the clinical use in pediatric-aged patients has increased steadily over time and, in 2024, sugammadex was approved for use in patients from birth to < 2 years old [8]. Recent work has demonstrated the relative safety and efficacy of sugammadex in this age group, but there are a limited number of large cohort studies to support these findings and there exist limited data in patients following surgery for congenital heart disease (CHD) [9–11].

While sugammadex has been shown to be highly effective at reversing neuromuscular blockade, adverse effects have been reported including bradycardia, hypotension, as well as anaphylactoid or allergic reactions [12–15]. In patients receiving large doses (16 mg/kg), a mild prolongation of the prothrombin and partial thromboplastin times has been reported; however, no clinically significant bleeding has been noted [13].

These adverse effects may be of particular concern in vulnerable populations, such as infants with CHD, who may be at a higher risk related to hemodynamic instability (bradycardia and hypotension) and pulmonary complications. When compared with neostigmine, sugammadex has been associated with a reduced incidence of postoperative pulmonary complications in adults and no difference in pediatric-aged patients [16, 17]. The primary study objective was to evaluate the perioperative adverse effects associated with the administration of sugammadex in pediatric patients ≤ 2 years of age with CHD. Secondary objectives included a comparison of these effects with patients receiving neostigmine and an evaluation of intraoperative monitoring practices for neuromuscular blockade.

## Materials and Methods

This study was approved by the Institutional Review Board (IRB) of Nationwide Children’s Hospital and was conducted in compliance with the ethical standards of the responsible institution on human subjects as well as with the Helsinki Declaration. As a retrospective study using only deidentified data, the need for individual informed consent was waived.

### Study design

The present study was a single-center, retrospective chart review conducted at Nationwide Children’s Hospital, a tertiary care children’s hospital in Columbus, Ohio. We adhered to applicable Strengthening the Reporting of Observational Studies in Epidemiology (STROBE) guidelines for cohort studies. Records were retrieved from the hospital electronic medical record (EMR) system (EPIC, Verona, WI). The study population included patients less than 2 years of age undergoing surgical procedures for the diagnosis, correction or palliation of CHD between January 2015 and October 2024 who received either sugammadex or neostigmine for reversal of neuromuscular blockade. The neostigmine group included patients cared for prior to the introduction of sugammadex into our operating rooms, as more than 99% of patients requiring reversal of neuromuscular blockade received sugammadex after its addition to our hospital pharmacy. Patients were excluded if they were more than 2 years of age or did not receive sugammadex or neostigmine for reversal of neuromuscular blockade.

The primary focus of this review was on dosing, efficacy, and the adverse effect profile of sugammadex in patients with CHD during the perioperative period. Demographic data extracted included patient age, height, weight, gender, and American Society of Anesthesiologists (ASA) physical status. Surgery and anesthesia information included total anesthesia time, surgery type and duration, and neuromuscular blockade reversal agent used, including sugammadex and neostigmine. Post-surgery and postoperative information included whether the patient’s trachea was extubated in the operating room and whether there was evidence of residual neuromuscular blockade following reversal agent administration. Information related to sugammadex or neostigmine included the dose (mg/kg) of the initial and any subsequent doses. Standard dosing based on the manufacturer’s recommendations include 2–4 mg/kg based on the train-of-four (TOF) response. Efficacy was assessed by the success or failure of tracheal extubation as well as evidence in the EMR of residual neuromuscular blockade. Adverse effects related to sugammadex or neostigmine included evidence of allergic/anaphylactoid reactions, hemodynamic or respiratory effects including bradycardia, bronchospasm, altered resistance/compliance, and other end-organ adverse effects. This included notes from the EMR as well as the need to administer rescue medications including vasoactive agents, anticholinergic agents, or albuterol to treat bronchospasm.

### Statistical analysis

A random cohort of patients was evaluated over the study period. The sample size was determined by data availability, and no *a priori* calculation was performed. Demographic and clinical characteristics are summarized using descriptive statistics. Categorical variables are presented as frequencies and percentages, and continuous variables as medians with interquartile ranges (IQR), given their non-normal distribution as assessed by the Shapiro-Wilk test.

Comparison between sugammadex and neostigmine groups were performed using the Pearson’s Chi-square test or Fisher’s exact test as appropriate, for categorical variables. Continuous variables were compared using the Wilcoxon rank-sum test. A two-sided P value < 0.05 was considered statistically significant. Data management and analysis were performed in Stata version 18 (StataCorp, College Station, TX).

## Results

### Characteristics of study population

A total of 501 encounters were identified for review during the study period. Four encounters were excluded; three for age ≥ 2 years and one for an implausible dose-to-weight ratio for neostigmine administration, yielding a final analytic cohort of 497 encounters across 375 unique patients. The median age of the cohort was 7 months (IQR: 3–12), and 54.1% were male. Most patients were classified as ASA physical status ≥ 3 (97.2%, n = 483). Cardiac catheterization was the most common procedure type (53.9%; n = 267), followed by other cardiac procedures without cardiopulmonary bypass (CPB; 22.0%; n = 109) (including pacemaker placement or shunt, reconstructive procedure, and unlisted cardiovascular procedures), cardiac surgery with CPB (21.0%; n = 104), and non-cardiac procedures (3.0%; n = 15). The median total anesthesia time was 268.7 min (IQR: 214.1–327.7).

Of the included encounters, 139 (28.0%) involved neostigmine administration and 358 (72.0%) involved sugammadex administration. Baseline characteristics were generally similar between groups (Table 1). Age, sex, height, weight and procedure type did not differ significantly (all P > 0.05). However, patients who received sugammadex were more likely to have ASA physical status ≥ 3 compared with those who received neostigmine (98.6% vs 93.5%; P < 0.01). Total anesthesia time was modestly longer in the sugammadex group (275.3 min; IQR: 218.5–334.2) compared to the neostigmine group (253.4 min; IQR: 185.7–314.6; P = 0.01), while total procedure time was similar between groups.

**Table 1:** Demographic and Clinical Characteristics of Study Population^a^

Characteristics	Overall	Neostigmine	Sugammadex	P value
Study population, n (%)	497	139 (28.0)	358 (72.0)	
Age (months)	7 (3,12)	6 (4,12)	7 (3,13)	0.73
Sex				0.88
Female	228 (45.9)	63 (45.3)	165 (46.1)	
Male	269 (54.1)	76 (54.7)	193 (53.9)	
Height (cm)	65 (58,73)	65 (57,73.3)	65 (58,73)	0.73
Weight (kg)	6.6 (5.1,8.8)	6.6 (5.1,9.3)	6.6 (5,8.7)	0.39
ASA classification				< 0.01
2	14 (2.8)	9 (6.5)	5 (1.4)	
≥ 3	483 (97.2)	130 (93.5)	353 (98.6)	
Surgical procedure				0.37
Cardiac catheterization	267 (53.9)	81 (58.7)	186 (52.1)	
Cardiopulmonary bypass	104 (21.0)	22 (15.9)	82 (23.0)	
Non-cardiac	15 (3.0)	4 (2.9)	11 (3.1)	
Other cardiac	109 (22.0)	31 (22.5)	78 (21.8)	
Total anesthesia time (min)	268.7 (214.1, 327.7)	253.4 (185.7, 314.6)	275.3 (218.5, 334.2)	0.01
Total procedure time (min)	174.8 (135.4, 222.8)	174.8 (128.9, 225)	172.6 (137.6, 220.6)	0.97

Data are presented as counts and percentages for categorical variables and as median (IQR) for continuous variables. ^a^All data are reported per encounters. P values were calculated using Pearson’s Chi-square test or Fisher’s exact test, as appropriate, for categorical variables and Wilcoxon rank-sum test for continuous variables. ASA: American Society of Anesthesiologists.

### Dosing

The median dose of neostigmine administered was 0.07 mg/kg (IQR: 0.06–0.07; mean ± standard deviation (SD): 0.07 ± 0.02), with a range of 0.01 to 0.20 mg/kg. The median initial dose of sugammadex was 4.13 mg/kg (IQR: 3.83–4.88; mean ± SD: 4.53 ± 1.98), with a range of 0.53 to 16.26 mg/kg. Most encounters involved a single administration of the reversal agent (88.5%; n = 440); however, multiple dosing was more frequent in the sugammadex group compared with the neostigmine group (14.5%; 52/358 vs 3.6%; 5/139; P < 0.001).

### Adverse effects

**Figure 1:**
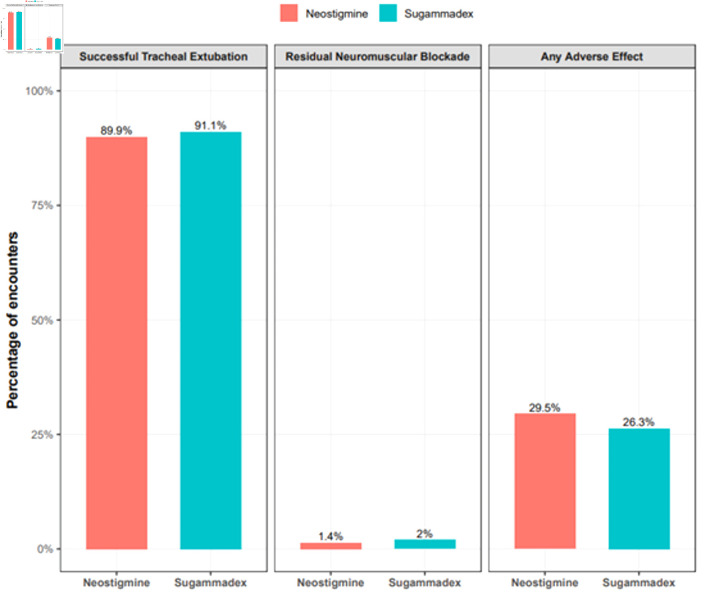
Rates of successful tracheal extubation, residual neuromuscular blockade, and adverse events by reversal agent. There was no statistically significant difference between any of the three outcomes when comparing sugammadex with neostigmine. Please see Table 2 and its footnote for a list of adverse effects. As this was a retrospective review, the temporal and causal association with sugammadex could not be determined

Perioperative adverse effects are summarized in Table 2 and Figure 1. There were no documented adverse events in most encounters, occurring in 70.5% (98/139) of neostigmine cases and 73.7% (264/358) of sugammadex cases. Bradycardia occurred at a low and similar rate in both groups (1.4%). Additional adverse events were categorized as “other” and occurred in 25.8% (n = 128) of encounters overall, with a similar distribution between groups (neostigmine 28.1%; sugammadex 24.9%). These events were heterogeneous and included a range of respiratory, cardiovascular, neurological, and vascular findings, which may or may not have had a direct causal relationship to the reversal agent. Respiratory events were most prevalent and included hypoxemia, increased work of breathing, respiratory insufficiency, laryngospasm, stridor, and tachypnea. Cardiovascular events included hypotension, hypertension, tachycardia, cardiac arrest, cardiogenic shock, complete heart block, and ventricular tachycardia. Neurological events included agitation, delirium, altered mental status, sedation, and seizure-like activity. Vascular events included limb ischemia, absent peripheral pulses, and arterial thrombus. No cases of anaphylaxis or anaphylactoid reaction were documented in either group.

**Table 2:** Measures of Efficacy and Adverse Effects

Characteristics	Overall	Neostigmine	Sugammadex	P value
Study encounter, n (%)	497	139 (28.0)	358 (72.0)	
Successful tracheal extubation				0.70
No	46 (9.3)	14 (10.1)	32 (8.9)	
Yes	451 (90.7)	125 (89.9)	326 (91.1)	
Residual neuromuscular blockade				1.00
No	488 (98.2)	137 (98.6)	351 (98.0)	
Yes	9 (1.8)	2 (1.4)	7 (2.0)	
Adverse effect				0.47
None	362 (72.8)	98 (70.5)	264 (73.7)	
Bradycardia	7 (1.4)	2 (1.4)	5 (1.4)	
Other	128 (25.8)	39 (28.1)	89 (24.9)	

Data are presented as counts and percentages. P values were calculated using Pearson’s Chi-square test or Fisher’s exact test, as appropriate. For statistical testing, adverse effect was analyzed as a binary variable (any vs none). Other adverse effects included respiratory dysfunction (postoperative respiratory insufficiency, hypoxemia, cough, increased work of breathing, stridor, congestion, hypoventilation, laryngospasm, hypercapnia, respiratory arrest, pleural effusion); hemodynamic concerns (arrythmias, hypotension, hypertension, decreased perfusion, increased lactate, tachycardia, heart block, bradycardia, altered peripheral pulses); central nervous system (delirium, agitation, decreased mental status, seizure activity, sedation); and miscellaneous issues (hyperglycemia, emesis, postoperative hemorrhage). As this was a retrospective review, the temporal and causal association with sugammadex could not be determined.

### Efficacy

Measures of efficacy are presented in Table 2 and Figure 1. Overall, successful tracheal extubation occurred in 90.7% (n = 451) of encounters and was comparable between groups (neostigmine 89.9%; sugammadex 91.1%). Residual neuromuscular blockade was documented in 1.8% (n = 9) of encounters overall, with similar rates in both groups (neostigmine 1.4%; sugammadex 2.0%). Quantitative TOF monitoring was not consistently documented in the EMR, which had the potential to limit the reliability of assessing residual neuromuscular blockade.

## Discussion

This retrospective cohort study describes the perioperative safety and efficacy profile of sugammadex compared to the historical use of neostigmine for reversal of neuromuscular blockade in infants less than 2 years of age with significant comorbid cardiovascular diseases including CHD. Across 497 encounters, sugammadex was generally well-tolerated in this vulnerable population, with a low and comparable incidence of bradycardia, a comparable distribution of other adverse events, and a non-inferiority rate of successful tracheal extubation. These findings are particularly noteworthy because patients in the sugammadex group carried a higher ASA physical status classification, which may increase the likelihood of perioperative adverse events. Most importantly, even in the setting of comorbid CHD and following surgery CPB, there was no increased incidence of hemodynamic or conduction concerns. Even with a higher overall ASA physical status profile, the adverse event profile and incidence was similar between groups, which descriptively supports that the safety of sugammadex is comparable to neostigmine.

Bradycardia has been recognized as a potential adverse effect of sugammadex since its introduction into clinical practice and is noted in the FDA package insert as a serious adverse event that may progress to cardiac arrest [12–15]. However, no definitive mechanism has been established. The incidence of bradycardia in the current study was 1.4% in both groups, which is consistent with prior reports demonstrating low rates of clinically significant bradycardia following sugammadex administration in pediatric patients [12, 15, 18]. However, when comparing the two reversal agents directly in pediatric populations, the literature remains mixed. Some studies report no significant difference in bradycardia incidence between neostigmine and sugammadex, while others demonstrate a lower incidence with sugammadex [19, 20]. The absence of any cases of anaphylaxis in our cohort is also reassuring, as hypersensitivity reactions represent another recognized adverse effect of sugammadex, particularly in patients with prior or repeat exposures [21–23].

Additional adverse events observed in the current study were heterogeneous and spread among respiratory, cardiovascular, neurological, and vascular categories. Respiratory events, including hypoxemia, increased work of breathing, respiratory insufficiency, laryngospasm, and stridor, were the most prevalent, and cardiovascular events included tachycardia, hypotension, cardiogenic shock, cardiac arrest, and complete heart block. The clinical context of these events is important to recognize when evaluating these findings and their potential causal relationship to either sugammadex or neostigmine. The majority of patients in this cohort underwent high-acuity cardiac procedures, including cardiac catheterization and surgery requiring CPB, and carried ASA classifications ≥ 3 in more than 97% of encounters. As such, many of these adverse events are likely attributable to the underlying CHD, associated comorbid conditions, and the hemodynamic and respiratory impact of the complex surgical procedure, intervention, or anesthetic care rather than to the agent used for reversal of neuromuscular blockade. Accordingly, these findings reinforce the importance of contextualizing adverse event data in this population carefully when attributing causality.

Successful tracheal extubation was achieved in over 90% of encounters overall, with comparable rates between groups. These findings are consistent with the existing literature demonstrating that sugammadex facilitates reliable and rapid reversal of neuromuscular blockade [24–26]. In the context of CHD surgery, early tracheal extubation has been associated with a reduced need for sedation and vasoactive agents, shorter intensive care unit (ICU) and hospital length of stay, and fewer complications of prolonged mechanical ventilation, demonstrating the value of effective neuromuscular blockade reversal even beyond the immediate perioperative period [27–29]. Given the growing body of evidence supporting fast-track tracheal extubation strategies in pediatric cardiac surgery, the ability to achieve rapid and reliable reversal of neuromuscular blockade is increasingly important. Although we noted no significant difference in our study cohort, sugammadex may theoretically play a role by allowing more predictable recovery and reducing the risk of residual blockade.

An unexpected yet notable finding in the current study was the wide variability in sugammadex dosing, with individual doses ranging from 0.53 to 16.26 mg/kg in the sugammadex group. This variability is consistent with the challenge of dosing sugammadex without objective information regarding the depth of neuromuscular blockade using quantitative neuromuscular blockade monitoring to guide dosing at the time of reversal. Recent guidelines, including those from the ASA and the European Society of Anesthesia and Intensive Care/European Society for Pediatric Anesthesia recommend quantitative TOF monitoring with documentation of a TOF ratio of ≥ 0.9 prior to extubation [30, 31]. In the present study, TOF monitoring (qualitative or quantitative) was not consistently documented in the EMR across either group, limiting the reliability to assess residual neuromuscular blockade and preventing the use of objective blockade depth to guide dosing decisions. The latter may explain the variability noted in sugammadex dosing as dosing recommendations (2 mg/kg versus 4 mg/kg) should be based on the TOF at the time of reversal.

This inconsistency in monitoring practice is clinically important, particularly in infants. The developing respiratory anatomy of infants along with the variable pharmacokinetics of NMBAs creates specific vulnerabilities related to residual neuromuscular blockade, including impaired pharyngeal function, airway obstruction, and decreased hypoxic ventilatory response [32, 33]. Surveys and quality improvement work involving pediatric anesthesiologists further indicate that routine quantitative TOF assessment is performed by only a minority of practitioners in the United States [34, 35]. Without objective information regarding the depth of neuromuscular blockade at the time of reversal, dosing decisions for sugammadex are imprecise. In the present cohort, this likely contributed to the wide range of administered doses. Underdosing sugammadex could increase the risk of inadequate reversal and residual neuromuscular blockade while higher doses may expose patients to unnecessary drug, resulting in increased cost without added benefit [36]. In a population already at increased baseline risk for perioperative respiratory adverse effects, such deviations may carry significant clinical consequences. The absence of standardized monitoring documentation in the current study is, therefore, both a study limitation and a clinical finding that mirrors practice patterns reported in the broader literature, highlighting a clear opportunity for quality improvement.

There are several limitations of the present study that warrant acknowledgment. First, this was a single-center, retrospective cohort study, which limits the generalizability of findings and introduces the potential for selection bias and problems with identification of the true incidence of specific adverse effects. The neostigmine group served as a historical comparator, reflecting a period prior to the departmental-wide adoption of sugammadex which occurred shortly after its addition to our operating room formulary. This design means that differences in the two groups may in part reflect trends in patient care practices and case mix over the study period rather than the effects of the reversal agent alone. Second, as noted, quantitative TOF monitoring was not consistently documented, which limits the ability to assess reversal and the true incidence of residual neuromuscular blockade in either group. Third, given the clinical complexity of this patient population, attribution of specific adverse events to the reversal agent is inherently uncertain, as many events may be explained by the underlying CHD, the surgical procedure, or anesthesia factors independent of the reversal agent. Finally, as a single-center study conducted at a high-volume pediatric center, practices and outcomes at this institution may not be representative of other centers with different experience in the perioperative management of infants with CHD.

In conclusion, in the current cohort of infants with CHD, sugammadex was associated with a low incidence of perioperative adverse effects and rates of successful tracheal extubation similar to neostigmine. These patterns held despite higher ASA status among patients receiving sugammadex, which suggests greater baseline illness severity. Observed adverse events were heterogeneous and likely reflect the underlying clinical context in addition to any reversal agent effects. This study also highlights practice variability, including inconsistent documentation of quantitative TOF monitoring, which limits the ability to assess residual blockade and may contribute to variable dosing of sugammadex. In this physiologically vulnerable population, standardized monitoring and prospective data would help clarify how to best dose sugammadex. Taken together, these findings support the continued use of sugammadex in infants with CHD while highlighting the limits of current evidence. Larger, prospective studies with consistent monitoring are needed to better define the safe use of sugammadex in this population and to more clearly separate drug-related effects from other perioperative contributions to adverse events.

## Data Availability

The data supporting the findings of this study are available from the corresponding author upon reasonable request.
